# Present Limits to Heat-Adaptability in Corals and Population-Level Responses to Climate Extremes

**DOI:** 10.1371/journal.pone.0024802

**Published:** 2011-09-20

**Authors:** Bernhard M. Riegl, Sam J. Purkis, Ashraf S. Al-Cibahy, Mohammed A. Abdel-Moati, Ove Hoegh-Guldberg

**Affiliations:** 1 National Coral Reef Institute, Nova Southeastern University, Dania, Florida, United States of America; 2 Biodiversity Management and Conservation, Environment Agency Abu Dhabi, Abu Dhabi, United Arab Emirates; 3 Environmental Assessment Department, Ministry of Environment, Doha, Qatar; 4 Global Change Institute and ARC Centre of Excellence for Coral Reef Studies, The University of Queensland, Brisbane, Australia; King Abdullah University of Science and Technology, Saudi Arabia

## Abstract

Climate change scenarios suggest an increase in tropical ocean temperature by 1–3°C by 2099, potentially killing many coral reefs. But Arabian/Persian Gulf corals already exist in this future thermal environment predicted for most tropical reefs and survived severe bleaching in 2010, one of the hottest years on record. Exposure to 33–35°C was on average twice as long as in non-bleaching years. Gulf corals bleached after exposure to temperatures above 34°C for a total of 8 weeks of which 3 weeks were above 35°C. This is more heat than any other corals can survive, providing an insight into the present limits of holobiont adaptation. We show that average temperatures as well as heat-waves in the Gulf have been increasing, that coral population levels will fluctuate strongly, and reef-building capability will be compromised. This, in combination with ocean acidification and significant local threats posed by rampant coastal development puts even these most heat-adapted corals at risk. WWF considers the Gulf ecoregion as “critically endangered”. We argue here that Gulf corals should be considered for assisted migration to the tropical Indo-Pacific. This would have the double benefit of avoiding local extinction of the world's most heat-adapted holobionts while at the same time introducing their genetic information to populations naïve to such extremes, potentially assisting their survival. Thus, the heat-adaptation acquired by Gulf corals over 6 k, could benefit tropical Indo-Pacific corals who have <100 y until they will experience a similarly harsh climate. Population models suggest that the heat-adapted corals could become dominant on tropical reefs within ∼20 years.

## Introduction

The all-important symbiosis between corals and symbiotic dinoflagellates is destabilized by environmental stress, particularly elevated sea temperatures [1], [2]. Corals turn white, i.e. bleach, as they lose their symbionts and become vulnerable to increased rates of disease and mortality. In combination with local stresses, increasing global stress is driving a global decline in coral populations [3], [4]. Outbreaks of mass coral bleaching and mortality are finely tuned (adapted) to the local climatology and have been used with climate projections to explore how coral communities are likely to change as the world warms [1]. The thermal threshold for mass coral bleaching and mortality across the majority of Indo-Pacific and Caribbean habitats lies around 30–32°C [1], [5]. Some populations of corals, however, experience extreme temperatures and are valuable in terms of understanding the outer envelope of genetic responses to climate change. Here, we present data on corals within the hottest sea with abundant coral on the planet (Arabian/Persian Gulf) that may provide thermally tolerant species and varieties for other tropical habitats as they warm. These corals survive levels of thermal stress that would probably kill even the most tolerant corals growing elsewhere. Here we analyze models of population dynamics to test whether under extreme disturbance scenarios, as are presently experienced by Gulf corals and expected on tropical coral reefs by 2099 [6], coral populations will increasingly fluctuate and will consequently be forced to abandon the present “reef-building” mode. We also investigate whether, if moved into the tropical Indo-Pacific by currents or assisted migration, today's Gulf corals would have the potential to be dominant and how long replacement of presently resident genotypes would take, given future disturbance scenarios.

## Results

### Large-scale thermal environment

**Figure 1:**
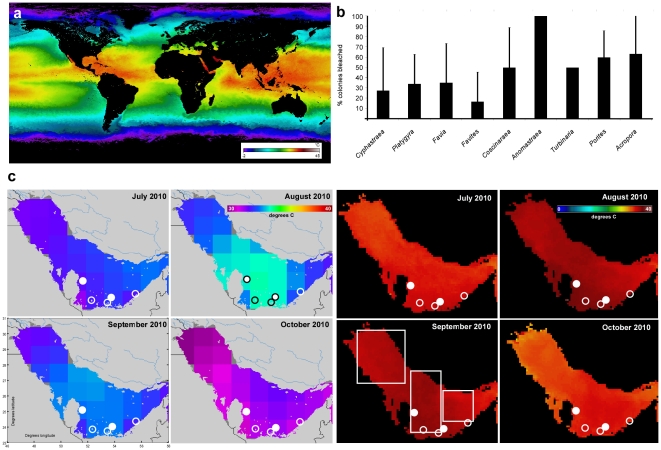
The 2010 bleaching event in the Arabian/Persian Gulf. (a) Modis SST composite for September 2010. The Gulf was the hottest part of the world's ocean. Conditions comparable to the Gulf will prevail throughout the tropics in 2099. (b) Mean (+standard deviation) bleaching incidence of the most common coral genera across SE Gulf coral monitoring sites (identified by circles in Fig. 1c) in October 2010, during regeneration from the main bleaching event. Bleaching was worst in *Anomastraea irregularis* (a rare species), *Acropora* spp. and *Porites harrisoni* (the most common framework-builders) (c) HadISST and Pathfinder (Modis) data showing the greatest heat in the central and SE Gulf. SST averages mentioned in the text were calculated from areas within the white boxes. Circles show monitoring sites (empty circle = coral monitoring only, full circle = coral monitoring plus temperature monitoring). Datasource: http://badc.nerc.ac.uk; http://www.nodc.noaa.gov.

In one of the world's hottest years, 2010 [7], the central and Southeast Arabian/Persian Gulf was the world's hottest sea and developed record sea temperatures that resulted in >60% of all corals bleaching by August–September (Modis, HadISST and HadISST2 data, Fig. 1a). A significant positive temperature anomaly of 1–3°C existed over most of the NW Indian Ocean, Red Sea, and Gulf region persisting into December 2010. Gulf-wide, the strongest heating was restricted to its central and SE parts (Fig. 1c). Highest sea surface temperatures were observed in August, with maximum heat in the Abu Dhabi and Qatar offshore islands and banks. Average monthly SST (Pathfinder data) for three large sampling regions (Fig. 1c) in August/September was 33.5°C in the northern Gulf, 32.78°C in the eastern Gulf, but 34.45°C in the central bleaching region. The far eastern Gulf received less heat. HadISST and Pathfinder datasets show that heat abated over October.

### Bleaching event

In August and September 2010, corals in the SE Gulf bleached by 60→80%, depending on site (Fig. 1b). This was a repeat of events in 1980, 1996, 1998, 2002 [10]. By late October 2010, bleaching had mostly abated and corals had recovered at all sites in Abu Dhabi, but significant mortality in *Acropora* (∼90% at Al Heel and Dalma, ∼50% at Ras Ghanada) was observed due to coral diseases (white syndrome) immediately after recovery from bleaching. Corals in Qatar recovered over November 2010. Faviids and poritids bleached strongly, but recovered with little (<10%) mortality. The least susceptible taxon was *Cyphastraea* with the highest proportion of unbleached corals in all localities (100–60%).

### Bleaching thresholds

**Figure 2:**
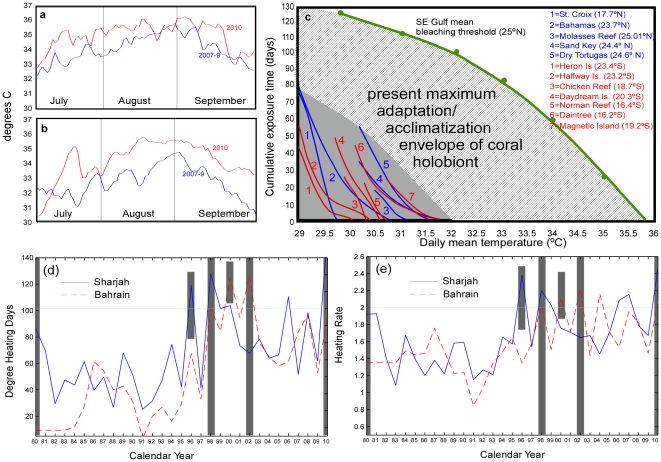
Water temperatures on reefs in the Arabian/Persian Gulf in 2010. (a) Abu Dhabi, (b) Qatar. (c) Bleaching thresholds for Gulf corals significantly exceed those of Indo-Pacific or Caribbean corals. These differences are a consequence of adaptation to the higher water temperatures of the Gulf. Climate change scenarios [6] suggest Gulf-like conditions throughout the tropics by 2090–99. (c) Annual Degree Heating Days and (d) Heating Rate calculated from air temperatures measured at Sharjah and Bahrain as proxies for bleaching. DHD and HR clearly increased over time. Known region-wide bleaching events are full-length grey bars, short bars for local bleaching.

Bleaching and mortality thresholds in 2010 derived from *in situ* temperature loggers were the highest ever recorded anywhere. Water temperature maxima measured on the reefs on an hourly (36.4°C, Sep. 9–10, Abu Dhabi) or daily basis (35.72°C, Aug. 15, Qatar) exceeded long-term summer means by ∼2.5°C (Fig. 2a,b). Corals were exposed to 33–35°C on average about twice as long as in non-bleaching years. The 2010 event showed that Gulf corals bleach when daily mean temperatures remain above 34°C for a total of ∼8 weeks (Abu Dhabi 67 days, Qatar 57 days) during which more than 3 wk are spent above 35°C (Abu Dhabi 23 days, Qatar 33 days; Fig. 2a, b). At the Qatar site, corals spent 8 days above daily mean temperatures of 35.5° and two days at the maximum 35.7°C, while at the Abu Dhabi site the maximum exposure was 3 days at 35.4°C. These levels are not only considerably higher than for corals within the Indo-Pacific and Caribbean generally, but the highest ever recorded [1], [5]. Long-term (3 month) mean summer temperatures were 33.71°C (Abu Dhabi, 4 m) and 32.83°C (Qatar, 5 m), exceeding Caribbean and Indo-Pacific bleaching thresholds by 1–2°C. This shows that Gulf corals can spend >3 months at temperatures that would immediately kill virtually all other corals [5].

Degree Heating Days (DHD) and Heating rate (HR) as indicators for bleaching were calculated from *in situ* data and air-temperatures (Table 1). Both DHD and HR of *in situ* records were higher at the Abu Dhabi site, which had a higher long-term mean summer temperature than the Qatar site and required only minimal increase for bleaching (Table 1). Indices from air temperatures coincided well with known disturbances and suggest DHD>100 in combination with HR>2 as a strong indication for bleaching in the Gulf (Fig. 2 d,e). Both indices, however, failed to predict the observed event in 2002, and suggested a possible event in 2007, which was not recorded (some local bleaching was observed, but not at the sampling sites). Thus, as is well known, local factors act to confound larger-scale predictions, which nonetheless remain reasonably accurate.

**Table 1:** Degree heating days (DHD) and heating rate (HR) at sites in Qatar and Abu Dhabi.

	DHD Jul.	HR Jul.	DHD Aug.	HR Aug.	DHD Sep.	HR Sep.
**Qatar**	21.4	0.9	74.3	2.4	40.66	1.35
**Abu Dhabi**	12.84	0.64	36.1	1.16	25.56	1.34

The peak in DHD in August suggests onset of bleaching in that month. DHD and HR are lower in Abu Dhabi since the site has ∼1°C higher long-term summer mean temperatures than Qatar, thus requiring less heat to reach the bleaching threshold.

### Repeat bleaching and population consequences

Local atmospheric and sea surface temperature records suggest overall warming over the past three decades. DHD and HR from air-temperature records at Bahrain and Sharjah showed a clear upward trend since 1980. The record's brevity does not allow definition of a pervasive trend, but suggests shorter recurrence of bleaching disturbances – a single bleaching event between 1980–1995 increased to four between 1995–2010. Local differences in bleaching years existed (UAE: 1980–1996–1998–2002–1010; Bahrain/Qatar: 1980?–1998–2000–2002–2010). Two near-bleaching events were observed 2002–10 that were avoided by lags in either DHD or HR (Fig. 2d,e). Corals in both temperature monitoring sites showed little regeneration after severe mortality in 1996/8, but regeneration at other sites monitored after that event was strong.

Recurrence of bleaching disturbances in the Gulf is already at levels predicted by [8] for the Great Barrier Reef in 2050 and heat levels are as predicted for the tropical ocean in 2099 [6]. The following questions arise: what trajectories will Gulf coral populations take in this environment and what are the implications for reef-persistence in general? And, more theoretically, how would the heat-adapted Gulf corals fare in comparison with their local congeners if they were to be transported, by ocean currents or human assistance, into the tropical Indo-Pacific?

**Figure 3:**
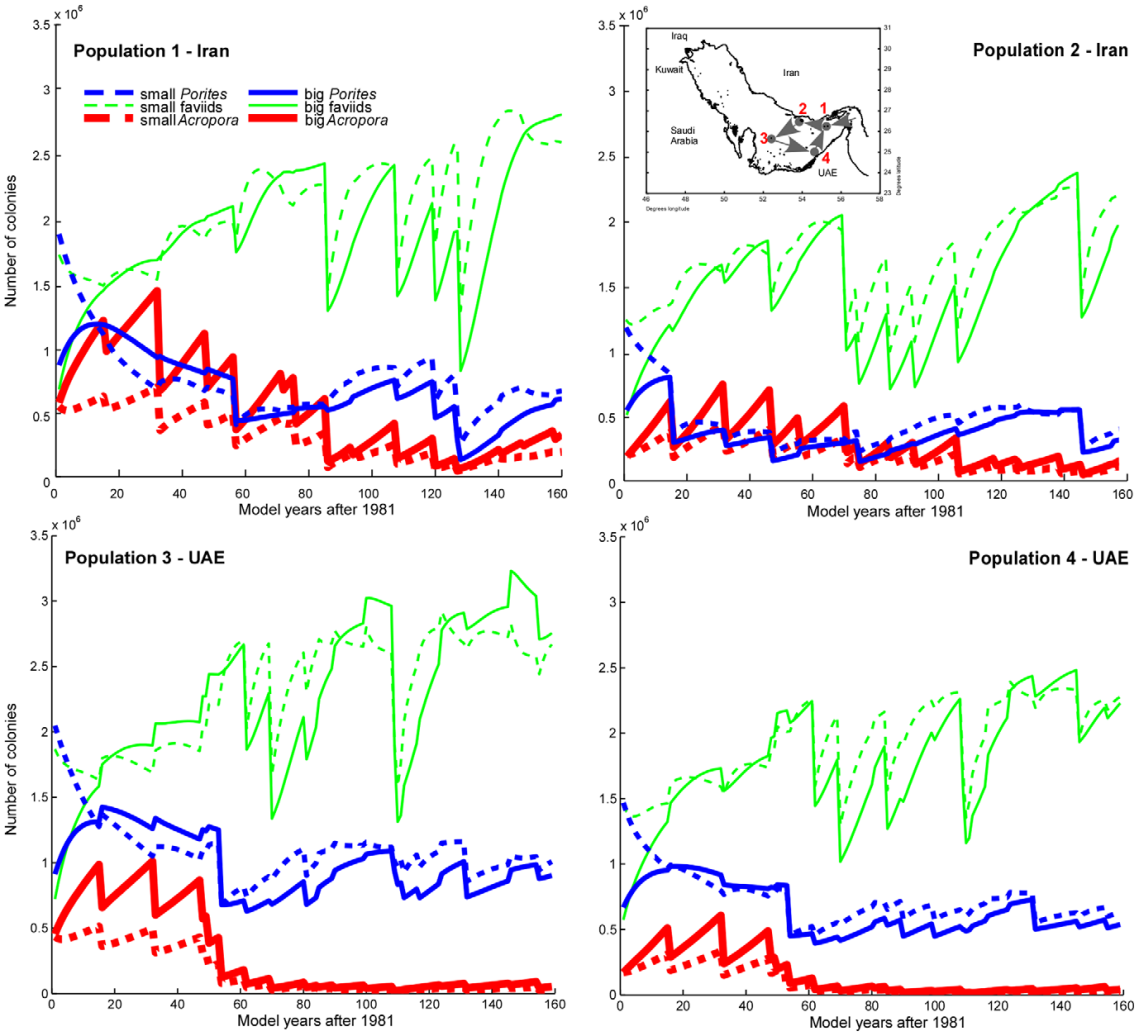
Population trajectories in four modeled Arabian/Persian Gulf coral populations. The situation reflects four connected populations. Two populations on Iranian offshore islands were modeled as connected to Halul (Qatar) and Ras Ghanada/Jebel Ali (UAE), where comparable dynamics was observed [10]. With increasingly closely-spaced bleaching disturbances, population trajectories become more erratic and reactive to connectivity pattern, suggesting stronger reliance on the metapopulation. Reef-building capability declines in step with the frequency of the dominant framebuilder, *Acropora*. Disturbance frequency from 1981–2010 as monitored in the field [10], thereafter randomized recurrence between 1–15 yr in Iran, 1–10 yr in UAE which has a higher disturbance frequency [10].

Population models show coral survival to be possible even under rapid disturbance recurrence if several connected populations exist (Fig. 3). Four populations were modeled in a scenario mimicking favored unidirectional movement, such as would be expected within the early summer cyclonic circulation in the eastern Gulf [9]. Fewer disturbances in cooler Iranian waters, combined with connectivity to reefs in the Straits of Hormuz [9], maintains bleaching-sensitive *Acropora* populations that have become severely depleted in the SE Gulf and only persist due to recruitment pulses arriving from Iran. Also the Iranian *Acropora* populations require subsidy from connected reefs. Thus, under these extreme conditions, reef corals resort to an increasingly “weedy” survival strategy, characterized by marked population fluctuations at any one site and increased reliance on the metapopulation as a repository of resilience. In the long term, however, this leads to an overall decline in frequency (Fig. 3). This strategy is most pronounced in the more bleaching and disease-susceptible taxa (*Acropora*, psammocorids), less in the more resistant taxa (poritids, faviids). Increasing the frequency of disturbance first causes a strong decline of *Acropora*, then increasingly of large *Porites*, and ends in communities mostly dominated by faviids. The highest disturbance frequencies result in populations of small faviids that form ephemeral patches (not illustrated). Such dynamics are no longer compatible with reef growth. Individual non-connected patches can only persist in model if recruitment assumptions are unrealistically high. Corals, at that point, are at best capable of forming non-rigid frameworks or no frameworks at all.

This leads to the following generalized conclusions: Increasingly frequent mortality disadvantages the major framebuilders: first *Acropora* then *Porites*, resulting in a faviid-dominated community; reef building will cease, corals will become sparse and metapopulations will fluctuate among patches.

**Figure 4:**
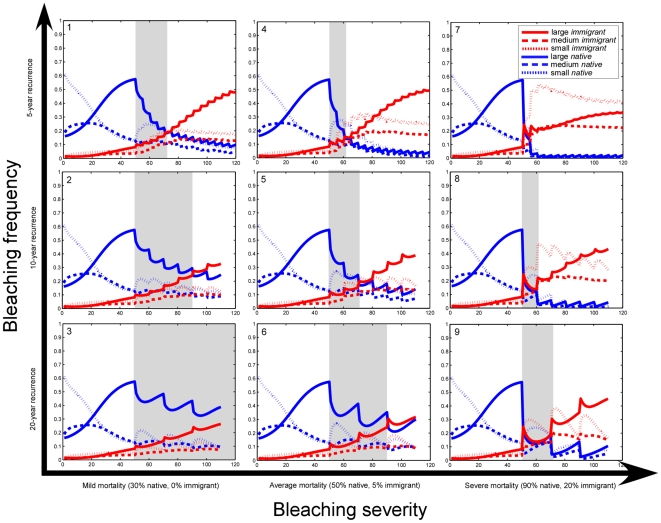
Time until dominance of immigrant species (started as 10% of resident population) under different disturbance scenarios. The model was started with an undisturbed 50-year interval to allow the native populations to equilibrate and was then regularly disturbed. Replacement times (grey bars) depend equally on severity or frequency of disturbance, equivalent effects shown by uniform decrease in replacement times along axes away from best-case scenario (#3). The tropical Indo-Pacific presently moves between scenarios #5, #6 and #9. Heat-adapted corals from the Gulf would become rapidly dominant and, in scenarios with more disturbance, they are the only option to maintain coral cover. Shapes of the curves change with variable carrying capacity and beginning population assumptions, but replacement trends are stable.

If, however, the heat-adapted Gulf corals were to be transported, by natural or assisted means, into the tropical Indian Ocean, the invading fauna would be spectacularly successful (Fig. 4). Over the next 50–100 years, the native less heat-adapted coral populations will suffer significant restriction during bleaching events that will not affect the corals from the Gulf (Fig. 4). The originally small introduced population (modeled as 10% of the resident population) would rapidly expand to dominate numerically and thus, even in the unlikely event that no interbreeding with the native populations occurred, the heat-adapted corals would rapidly dominate. However, if no further adaptation was to take place, then even these heat-resistant corals are likely to suffer increasing declines with even more rapid recurrence of even higher thermal extremes after 2100. At the very least, coral communities on tropical reefs might have been retained by 50–100 years beyond the likely extinction of the present assemblage.

### Level of threat to Gulf and tropical corals

The capability of Gulf corals to survive repeat disturbances was verified by theory and in the field [10], but recurrences as observed between 1995–2010 endanger at least the formerly dominant *Acropora*. This situation is worsened by mortality from disease during recovery from bleaching, as observed in 2010. Region-wide repeat mortality of *Acropora* (SE Gulf: 1996, 1998, 2000, 2002, 2010, NE Gulf: 2007, 2008) has severely depressed overall population levels and, in combination with rapidly recurring thermal and local man-made disturbances, makes full recovery of Gulf populations prior to the next bleaching mortality highly unlikely. Models confirm that bleaching recurrence at levels 1995–2010 would cause near or total extinction of *Acropora*, with first a shift to *Porites* and then faviid dominance (Figs. 3, 4). Long-term, Gulf corals are also under threat due to reduced hardground production caused by ocean acidification [11]. However, the same Gulf corals transplanted into the tropical Indo-Pacific, would prove immune to bleaching mortality for the rest of the century while local corals, at bleaching thresholds as observed today, would not be able to sustain populations (Fig. 4). Highest recorded temperatures from the tropical Pacific are hourly peaks of ∼34°C [12], which is far below the 8 weeks at >34°C needed to bleach Gulf corals.

## Discussion

The corals of the Arabian/Persian Gulf reveal the highest documented temperature tolerance of the coral holobiont. Tropical sea temperatures are projected to increase by at least 1–3°C above today's temperatures by the end of the century, resulting in temperatures throughout the tropics that are comparable to the Gulf today [6]. While Gulf corals were subjected to this climate for ∼6 ky [13], other corals will have <100 yrs to adapt. Current evidence suggests that adaptation within coral populations at these rates is unlikely to occur naturally, notwithstanding whether heat-resistance is due to adaptation of the symbionts or the coral animal. Gulf corals already have heat-tolerant symbiont populations (primarily clades C and D, [14], [15]).

Human intervention through assisted colonization may therefore represent an effective management tool [16]. Gulf corals appear likely to withstand the next hundred years of climate change in the tropical Indo-Pacific and are increasingly endangered in their current range over both short [17] and long terms [11]. Rampant coastal development has already physically removed many reefs in the region, lethal red tides have become increasingly common and have caused large-scale reef mortality [17], and ocean acidification will further stress coral reefs, compromise resilience, and in the long-term even reduce available hardgrounds [11]. Added to this, temperatures in the Gulf are rising [18] (Fig. 2) and extreme heat events are increasingly frequent [19], suggesting that bleaching events will also be more frequent and more severe. The WWF considers the Gulf ecoregion as ‘critically endangered’. *In situ*, the threat level for Gulf and tropical Indo-Pacific corals is comparable. Little can be done to ameliorate the situation for Gulf corals and important genetic adaptation that could help corals survive a changed climate might thus be lost. Assisted migration would therefore amount to *ex-situ* conservation of valuable heat-adapted Gulf stock (both coral animal and zooxanthellae) that, by potentially speeding-up thermal adaptation, would at the same time be beneficial for *in-situ* conservation of the recipient Indo-Pacific populations.

**Figure 5:**
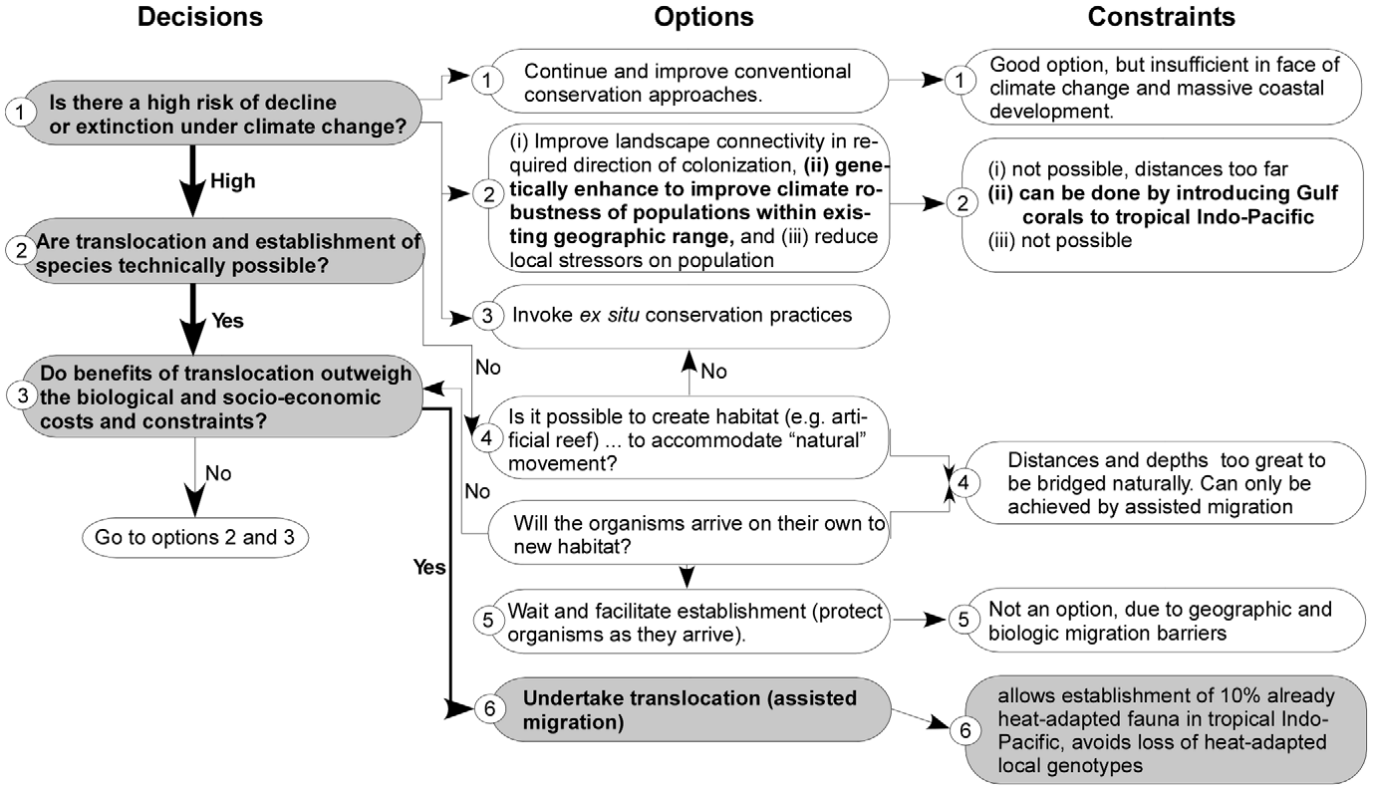
Decision framework for assisted migration modified and appended from [**16**] which clearly indicates that Gulf corals are prime candidates.

Assisted migration of Gulf corals to the tropical Indo-Pacific is suggested by existing decision frameworks for assessing possible translocations [16] (Fig. 5). Gulf species represent ∼10% of the common Indo-Pacific coral fauna and consequently represent an important heat-resistant core of ∼10% of species. In particular populations of *Acropora* have already been lost from the tropical Pacific due to lack of heat-adaptation and thus the re-introduction of heat-adapted genotypes may hold little genetic risk [16]. Latitudinal and altitudinal clines in thermal adaptations are common in other taxa [16]. While an anathema to some, considering these uniquely heat-adapted corals for assisted colonization with appropriate precautions may represent at least one option for preserving part of the biodiversity of coral reefs.

## MateriaIs and Methods

### Temperature data

Study locations were situated in the SE Gulf (UAE and Qatar). Bleaching thresholds were derived from temperature time-series 2007–10 at Bu Tinah, UAE (hourly, 4 m depth) and Fasht el Hurabi, Qatar (hourly, 5 m) obtained with VEMCO Minilog dataloggers with 0.01 degrees sensitivity. For Gulf-wide analysis of temperature anomaly, the HadISST (1×1° grid) and Pathfinder (1 km grid), and for worldwide analysis the HadISST, HadISST2 (5×5° grid; www.badc.nerc.uk), and MODIS datasets were used (obtained from neo.sci.gsfc.nasa.gov). Air temperature data for Sharjah and Bahrain International Airports (∼130 and ∼240 km from sampling sites), the closest stations to the coral monitoring sites, were obtained from http://climexp.knmi.nl. Air temperature data from Sharjah and water temperatures from Bu Tinah, as well as those of Bahrain and Fasht el Hurabi were highly correlated (R^2^ = 0.90). Also Degree Heating Days (DHD [21]) calculated from air temperatures correlated highly with those calculated from water temperatures (R^2^ = 0.93). Therefore, air temperatures were used to calculate DHD for the period 1980–2007 in which no *in situ* water temperatures were available.

### Bleaching thresholds

Time-integrated bleaching and mortality thresholds were derived for a strong (level 4 [5], [20]) bleaching event that occurred in the Arabian/Persian Gulf in September/October 2010. Days spent at 30–36°C average daily temperature were calculated. It is known from previous bleaching events that temperatures need to exceed 35.5°C for significant mortality to occur in the Gulf [10]. Also mortality curves [5], specific to the most susceptible genus *Acropora*, were derived. T_L50_, the temperature value at which 50% mortality occurs, is situated halfway between the bleaching threshold and the value causing 100% mortality (2010 in some study sites). We calculated degree heating days (DHD) and heating rate (HR) as stress indicators [21]. Long-term mean summer temperature was calculated from 1 July–30 Sep. 2007–2009 and T_heating_ as the daily average temperature during bleaching (1 July–30 Sep. 2010).

### Monitoring of bleaching mortality

At Ras Ghanada, Al Heel, Dalma and Bu Tina in Abu Dhabi and Fasht el Hurabi in Qatar (Fig. 1), phototransects or line transects were taken during and after the bleaching event. At Fasht el Hurabi, pre-existing phototransects, as well as tagged coral colonies were re-surveyed. All work took place at the end of the bleaching event (21 Sep.–10 Oct. 2010). Recovery was again monitored in January and June 2011. Monitoring of corals after previous bleaching events has been undertaken since 1995 (Ras Ghanada/Jebel Ali), 2004 (Fasht el Hurabi), and 2005 (Al Heel, Dalma and Bu Tinah).

### Models of coral population response

To simulate coral population dynamics, we used a three life-stage extension of a model by [10] with mortality, immigration/emigration terms for stepwise manipulations of local sensitivity analyses (details in [10]). Externally driven crises were forced by reducing the numbers of juvenile and/or adult corals as a function of disturbance at pre-determined time-intervals (bleaching events). Non-catastrophic mortality (diseases, predation, etc.) could be scaled according to climate scenarios. The model assumed that all corals started small and reproductively inactive and grew according to a fixed ratio into medium, then large colonies. This introduced a lag of 1 timestep in the rate of change of reproductively active corals. Medium and large colonies could reproduce sexually and asexually, but large colonies were assumed to be twice as fecund as medium sized [22]. Sexual recruits differed from asexual by their ability to migrate between populations, and coral populations could be self-seeding and/or receive larvae from, and donate to, connected populations. Recruits were not allowed to settle where another large coral already existed, but they could settle into the same cell as the recruit of another species [23]. Carrying capacity (*K*) was fixed with the effect of limiting recruitment by presence of existing corals [24]. Bleaching (multiplication factor <1 after a certain number of time-steps equivalent to years between disturbance) was made to affect large more than small corals [25]. Output of numerical frequency of each life stage was normalized to frequency of all stages in all species (0–1).
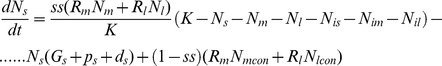
(1)

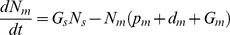
(2)

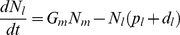
(3)
*N* is number of colonies in each stage, *K* carrying capacity (total number of individuals allowed without negative growth-rates), *G* a growth constant (small colonies growing into large ones), *d* and *p* mortality constants (disease, predation), *c* a competition constant, *ss* the self-seeding constant, 1-*ss* scales transfers to and from connected populations. Native and immigrant corals were given identical dynamics but different parameter values. Subscripts *s*, *m* and *l* refer to small, medium and large corals of either stock (either immigrants or native; immigrants = subscript *i*; the latter were only relevant if transplants into an existing population were considered). Subscripts *mcon*, *lcon* refer to large or small corals in a connected population that was modelled with the same equations 1–3 (*m* substituted by *mcon*, *l* by *lcon*).

In eq. 1, rate of change in abundance of small corals is a function of fertility of the medium and large corals (*RN*). Settlement is into free space only (medium and large corals to be subtracted from total carrying capacity). Gamete survivability and post-settlement fate is included in *R*. Losses occur by growth into larger size class (*G*), predation or disease (*p*, *d*). Self-seeding term *ss* determines ratio of local/imported propagules. In eq. 2 and 3, rates of change of medium and large corals depend on availability of smaller colonies, growth rate into the next size-class, and death by disease (*d*) or predation (*p*).

Three guilds (*Acropora*, poritids, faviids) were modeled (Fig. 3). Population sizes in the Gulf model were estimated from satellite imagery ground-truthed with photo-transects, from which also growth and recruitment parameters were obtained. Details in [10]. For this study, the model was tuned to the mortality frequencies observed at the sampling sites and as suggested by DHD and HR of atmospheric temperatures. Replacement dynamics of native and immigrant species (Fig. 4) using the same model assumed a constant reef area with populations of 4000 small, 1000 medium and 1000 big native corals and 0 small, 100 medium and 100 big immigrant corals. Populations were allowed to reach carrying capacity and were then disturbed with constant mortality at variable frequency and vice versa. Numerical approximations used second-order Runge-Kutta methods. Sensitivity analysis of a similar model was presented in [10] and not repeated here. Model outcomes were most sensitive to spacing of mass mortality and levels of recruitment.
